# Ex Situ Management and Reproduction of the Rediscovered Yellow-Spotted Bell Frog, *Ranoidea castanea*

**DOI:** 10.3390/ani15233404

**Published:** 2025-11-25

**Authors:** Michael S. McFadden, Loz Hush, Gemma Chaudhuri, Delvena Leong, Adam Skidmore, Aimee J. Silla, David A. Hunter

**Affiliations:** 1 Taronga Institute of Science and Learning, Taronga Conservation Society Australia, Mosman, NSW 2088, Australia; 2 Environmental Futures, School of Science, University of Wollongong, Wollongong, NSW 2522, Australia; 3 NSW Department of Climate Change, Energy, the Environment and Water, Albury, NSW 2640, Australia

**Keywords:** conservation, ex-situ, captive breeding, zoo, bell frog, conservation breeding program, threatened species, reproduction

## Abstract

The Yellow-spotted Bell Frog, *Ranoidea castanea* is a critically endangered species, native to the tablelands of New South Wales, Australia. It was rediscovered in 2009, after it had seemingly disappeared thirty years earlier. Upon rediscovery, a conservation breeding program was established at Taronga Zoo in Sydney, Australia, to secure an insurance population of the species. This paper describes the ex situ management and first successful captive breeding of this species, documents its life history data, and provides valuable learning that may benefit other conservation breeding programs. Although initial breeding attempts were unsuccessful whilst founding animals were housed indoors or in small outdoor enclosures, success was achieved between 2017 and 2019, once frogs were moved into a large outdoor enclosure (five clutches in three seasons). The attempted breeding of F1 individuals in an indoor dedicated facility in 2024 had greater success, with 60% of paired females laying eggs (six clutches in one season). Breeding behaviour occurred primarily between October and December, with clutches of unpigmented eggs laid amongst submergent vegetation from late October to mid-November. Overall, the program has resulted in the production of 2162 juvenile frogs for release, in addition to the retention of over 100 frogs for the insurance population. Herein, we provide important life history data for our study species and a framework for conservationists to propagate this species in an ex situ environment. We also highlight other key considerations, such as i) the importance of quickly establishing conservation breeding programs for threatened species and not assuming a targeted species will breed as readily as an analogue species, and ii) underscore the importance of undertaking baseline disease screening in the in situ environment.

## Simple Summary

The need for amphibian conservation breeding programs has dramatically increased in recent decades with the rapid decline and loss of species. This study describes the establishment of the conservation breeding program for the critically endangered Yellow-spotted Bell Frog (*Ranoidea castanea*), detailing the breeding challenges and success, early life history parameters, and highlighting the importance of program considerations such as immediate intervention and disease surveillance.

## 1. Introduction

Amphibians are the most threatened class of vertebrates, with approximately 40 percent of species currently under threat of extinction [1,2]. The rate of amphibian decline has escalated in recent decades due to a number of threats, including habitat loss and fragmentation, disease, climate change, overexploitation, and invasive species [2]. A single disease, the amphibian chytrid fungus (*Batrachochytrium dendrobatidis*), is thought to be responsible for the decline of over 500 species and the extinction of up to 90 species worldwide [3]. To prevent further losses, the establishment of conservation breeding programs for highly threatened species have been identified as a key conservation action [4]. Such programs are not only important to secure the species against short-term extinction, they provide an avenue for producing large numbers of offspring for translocations, conservation research, or other additional purposes, including community education programs. During the last two decades, the number of critically endangered species secured in insurance populations within zoos and aquariums has rapidly increased to address this need [5,6,7].

The Yellow-spotted Bell Frog *Ranoidea castanea* is a large terrestrial frog within the Australo-Papuan hyloid family Pelodryadidae, consisting of 233 treefrog species exhibiting wide morphological and life-history trait variation [8]. *R. castanea* was formerly found on the tablelands of northern, central, and southern New South Wales, in south-eastern Australia. The species experienced a rapid and widespread decline during the late 1970s and was thought to have disappeared by 1980. Due to the timing and pattern of this decline, its disappearance is indicative of the amphibian chytrid fungus (*B. dendrobatidis*) being the primary driver, which has also heavily impacted closely related species [9]. For three decades, it was assumed that this species was potentially extinct until its rediscovery in 2009 in a slow-moving creek in the Southern Tablelands region of New South Wales. The species is currently listed as critically endangered nationally under the Environmental Protection and Biodiversity Conservation (EPBC) Act and under the IUCN Redlist [10].

**Figure 1:**
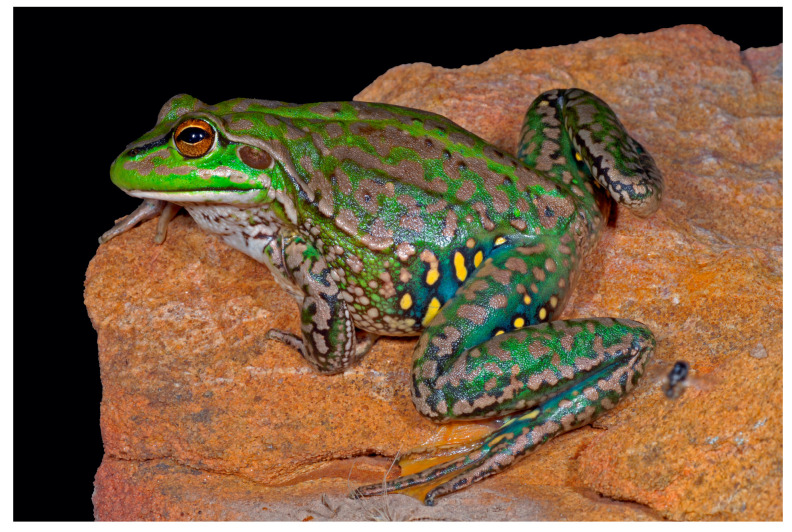
The Yellow-spotted Bell Frog (*Ranoidea castanea*). Photograph courtesy of David Hunter.

Similar to other members of the recently revised *Ranoidea* genus (consisting of five large terrestrial Bell Frog species) [8], *R. castanea* has predominantly green and golden colouration and grows to between 50 and 85 mm (Figure 1). It is distinguished from other members of the species group by large yellow spots in the inguinal region and on the rear of the thighs [11]. When observed in the wild, the species occupied ponds, wetlands, and slow-moving waterways with an abundance of aquatic and submergent vegetation. However, knowledge of the reproductive biology of this species prior to the decline was limited to two observations and assumed to be similar to the closely related Southern Bell Frog (*R. raniformis*) [12]. A recent taxonomic revision demonstrated its very close relationship with the newly defined northern subspecies *R. r. raniformis*, suggesting that they could possibly form one species [13]. However, the study stated that further taxonomic clarification would first be required to confirm the level phylogenetic divergence between *R. r. raniformis* and *R. castanea*, and to determine the genetic structuring of the northern and southern populations of *R. castanea*, with the northern populations not being detected in the wild for forty-five years [13].

Soon after the rediscovery of *R. castanea*, a rapid response was initiated to establish a conservation breeding program in partnership between the NSW Department of Climate Change, Energy, the Environment, and Water and Taronga Conservation Society Australia. Herein, we describe the ex situ management and reproduction of *R. castanea* at Taronga Zoo.

## 2. Materials and Methods

### 2.1. Study Animals

To establish the conservation breeding program, the collection of founders was initiated immediately after rediscovery along the four-kilometre section of creek that this species was found to inhabit [14]. The creek passed through open pastoral land on a private farming property north of Yass in New South Wales. Fourteen tadpoles and metamorphosing frogs were collected in 2010. The early life stage was targeted for collection to minimise the impact on the existing wild population. The closely related *R. raniformis* produces an average of approximately 3400 eggs [15]; thus, collecting larvae was predicted to have minimal impact. Despite obtaining a scientific license to collect up to 80 individuals and intensive searching, only fourteen specimens could be located. In subsequent years, summer flooding events associated with the La Nina weather pattern resulted in reduced breeding or larval survival, with no additional individuals located during 2011 and only one located and collected in 2012. Four additional metamorphosing frogs were collected in 2014. After this time, no further wild breeding has been observed and, despite repeated surveys of the site and the surrounding catchment in subsequent years, the species can no longer be located in the wild.

In 2015, a small population of adult *R. r. raniformis* were collected from near Colleambally in southern NSW. Due to the close genetic relationship between these species, individuals were collected and maintained for possible hybrid breeding with *R. castanea*, if breeding attempts within this species were unsuccessful and there were insufficient numbers of either sex of *R. castanea* remaining for continued attempts. If required, this would enable first-generation backbreeding to *R. castanea* to permit the retention of *R. castanea* genes.

### 2.2. Housing and Husbandry

#### 2.2.1. Indoor Enclosures

**Figure 2:**
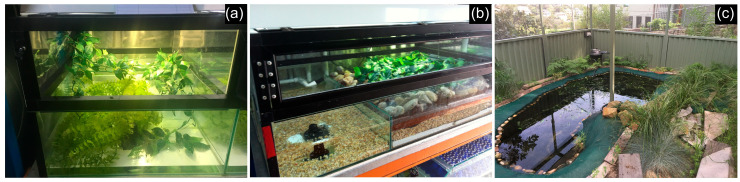
Housing for *R. castanea*. (**a**). The ‘standard indoor enclosure’ typically utilized for housing and breeding *R. castanea* in the Bell Frog conservation facility. (**b**). The ‘large indoor enclosure’ within the conservation facility. (**c**). The ‘large outdoor enclosure’ where successful breeding was first achieved.

From 2010 to late 2012, the frogs were housed within an isolated, quarantined room before being moved to a newly built Bell Frog conservation facility in July 2013. This facility is a refurbished, insulated shipping container dedicated to this species. Prior to the present study, this species had never been kept or bred in captivity, and husbandry guidelines for the species had therefore not been established. As a consequence, the initial enclosure design and husbandry methods adopted were determined based on the conditions successfully utilized to house and breed the closely related Green and Golden Bell Frog (*R. aurea*) [16]. These enclosures (‘standard indoor enclosure’) are designed in rows of three, each measuring 48 cm × 60 cm × 40 cm tall. They consist of a glass base, measuring 20 cm high, with a 20 cm high hood on top, constructed with a frame of hollow aluminium tubing and fine-gauge stainless steel mesh (Figure 2a).

The enclosure’s base is sloped toward the front of the enclosure, creating a land and water area. A drain is positioned at the front with a stem pipe inside to allow variable water depth. An 18 cm wide glass shelf with an angled ramp at the rear of each enclosure allows the frogs the opportunity to bask closer to the lights and exit the water when the water level is raised. Enclosure furnishings, consisting of artificial plants and PVC half-pipes, are artificial to allow for easy sterilization and prevent the accidental introduction of pathogens. Each enclosure has water slowly flowing through it to a sump below, passing through mechanical and biological filtration and a UV steriliser. A filtration bag containing activated carbon is also maintained in each sump. Prior to entering the facility, all water entering the facility passes through a sediment and activated carbon filtration system, with cartridges replaced every 4 months, and a typically maintained pH of 7.1–7.4. Total hardness (as CaCO_3_) is maintained between 55 and 70 mg/L, calcium at 13–16 mg/L, chlorine at <0.02 mg/L, and ammonia and nitrite levels at 0 mg/L.

In addition to the standard indoor enclosures described above, the Bell Frog conservation facility also contains larger breeding enclosures (‘large indoor enclosure’) (Figure 2b). These are 100 cm × 60 cm × 40 cm tall. These have glass bases with an aluminium and mesh lid, each 20 cm in height. A land area occupies 63 cm × 60 cm of the enclosure, with a substrate of smooth stones and artificial vegetation above a layer of cocopeat with a gravel base. The pool area of the enclosure occupies the remaining 37 cm × 63 cm, with a depth of 13 cm. Artificial aquatic vegetation was added to the water, and the base has a layer of aquarium gravel. Frogs were maintained in pairs in either enclosure type throughout the potential breeding season from late September to late March. Outside of the breeding season, frogs were maintained in single-sex groups in standard indoor enclosures.

Lighting for both the standard and large indoor enclosures was provided with two Reptisun^®^ 10.0 UVB T5 fluorescent tubes (Zoo Med Laboratories Inc., San Luis Obispo, CA, USA) positioned on top of the hood. The photoperiod was controlled by a HPM light-sensitive switch (Legrand Australia, Prestons, NSW, Australia) with a relay installed, resulting in the lights functioning from sunrise to sunset. This permitted lighting to closely reflect the natural photoperiod of the Sydney region. Additionally, as this species is partially diurnal and basks, a Reptisun^®^ 100W UVB mercury vapor bulb (Zoo Med Laboratories Inc., San Luis Obispo, CA, USA) was positioned above each enclosure and programmed by an electrical timer to come on daily for a two-hour block in both the mid-morning and late afternoon.

#### 2.2.2. Outdoor Enclosures

In 2013, a suspended outdoor enclosure was constructed in order to facilitate breeding attempts in an outdoor environment. The enclosure was 250 cm × 120 cm and 110 cm high. The rear panel of the enclosure and the rear 50 cm of the side and roof were solid steel sheets, whilst the front panel and front 70 cm of the roof and side panels were stainless steel mesh. The outdoor enclosures were suspended 70 cm above the ground on legs, each with perspex disks around them to prevent local frogs from climbing on top. The outdoor enclosure had a substrate of mixed cocopeat and sand above a gravel base. The top substrate layer was smooth stones, with a variety of artificial vegetation and sections of PVC pipe on the surface and partially buried to provide shelter. The outdoor enclosure had two ponds in the form of large food-grade plastic tubs. The larger tub was 126 cm × 70 cm × 30 cm depth with an operating volume of 210 L, whilst the smaller tub was 63 cm × 40 cm × 23 cm depth with an operating volume of 50 L. At each end of the enclosure, a PowerSun^®^ 160 W UVB mercury vapor lamp (Zoo Med Laboratories Inc., San Luis Obispo, CA, USA) was suspended to provide additional basking opportunities.

In December 2015, the remaining five males and two females were moved to a new large outdoor enclosure facility, purposefully built for this species. As there were only two females remaining at this time, and as the potential risk of the population declining further without breeding was high, they were accompanied by eight adult female *R. r. raniformis*. The enclosure measured 5.8 m × 4.7 m × 2.3 m high (Figure 2c). The walls, up to 1 m high, were constructed of smooth aluminium fencing and the roof and upper sides were enclosed with fine HDPE monofilament mesh with gaps of up to 5 mm. Such fine mesh was chosen to prevent access and the accidental introduction of disease or pathogens by other frog species. A large, oval-shaped pond was created, measuring 3.8 m in length and 1.8 m at the average width. The pond depth gradually increased across the pond from 10 cm to 40 cm in the deepest section. The pond was estimated to hold approximately 1700 L of water and underwent filtration and UV-sterilization with a Bioforce Revolution 6000 filter and Aquaforce 4000 submerged pump (Hozelock Australia, Keysborough, VIC, Australia). A small water change of less than five percent was undertaken every two weeks through the backwashing of the filter. A variety of pondside and low-cover vegetation was planted to provide cover but not excessive shade.

#### 2.2.3. Temperature and Diet

Between 2010 and 2013, whilst the frogs were housed in the conservation facility, room temperature was maintained at day and night settings of approximately 28 °C and 20 °C, respectively, whilst this was gradually lowered to day and night settings of approximately 15 °C and 8 °C during winter. From late 2013 until 2022, adult *R. castanea* were primarily housed in outdoor facilities, in either the large outdoor enclosure or the suspended outdoor enclosure, unless an individual required treatment. As Taronga Zoo is in a relatively similar climatic zone of south-eastern Australia as the wild population, additional heating was not required in the outdoor enclosures, though two basking lamps were positioned in the suspended outdoor enclosures to increase basking opportunities. From 2022 onwards, all frogs were returned to the indoor conservation facility.

Frogs were fed two to three times per week on a diet consisting predominantly of house crickets (*Acheta domestica*). The crickets were fed on a diet of Wombaroo Insect Booster (Wombaroo Passwell, Glen Osmond, SA, Australia). Prior to feeding, the crickets were dusted with Repashy^®^ Calcium Plus (Repashy Ventures Inc., Oceanside, CA, USA). Sub-adult to adult frogs were fed adult crickets, whilst juvenile frogs were fed crickets that were approximately the size of the width between the eyes of the frog.

## 3. Results

### 3.1. Reproduction

From the 2011/12 to the 2016/17 seasons, breeding attempts were unsuccessful in both the indoor and outdoor enclosures, despite utilising a number of techniques at the zoo that have demonstrated repeated success for the closely related *R. aurea*. The additional triggers employed in an attempt to elicit breeding activity consisted of (i) the use of a call playback of the male advertisement call throughout the night, (ii) spray systems engaged intermittently to simulate rain events, and (iii) abrupt increases in facility temperature. Calling was recorded in five of the six seasons, during the two-month period between 21 October and 31 December (Table 1). Only one infertile clutch was laid in the 2015/16 season in a suspended outdoor enclosure. Despite the lack of breeding success, amplexus was recorded on ten occasions throughout the initial four breeding seasons. These failed attempts were undertaken within the standard indoor and large indoor enclosures within the conservation facility, the suspended outdoor enclosure, and, during the first season, in the large outdoor enclosure.

Within the large outdoor enclosure, calling behaviour closely matched that observed indoors, with calling documented from 20 October to 27 December. Two fertile clutches were first laid in November 2017, followed by a further clutch in November 2018 and two more in October/November 2019 (Table 2). All five clutches consisted of unpigmented eggs laid amongst dense, submerged, fibrous roots within the pond. Due to the clutches being entangled amongst submerged vegetation, an accurate count of the egg number was not possible, though the two clutches laid in November 2017 were almost fully counted and were estimated to be 1500 ± 100 eggs and 1800 ± 100 eggs.

Tissues from the offspring of the late 2017 and late 2018 clutches were genotyped by the Australian Museum, utilising eleven microsatellite loci to determine the maternal parent [10] (further details are available on request, see the data availability statement below). During both years, there were two *R. castanea* and eight *R. r. raniformis* females in the outdoor enclosure. Testing revealed that the clutch in 2018 was laid by a *R. castanea* female, whilst of the two clutches laid in 2017, one was laid by a female of each taxa [17]. This signifies that 50% (2/4) of the potential breeding opportunities within *R. castanea* were successful, whilst only 6.25% (1/16) of the breeding opportunities were successful with *R. r. raniformis* females.

**Figure 3:**
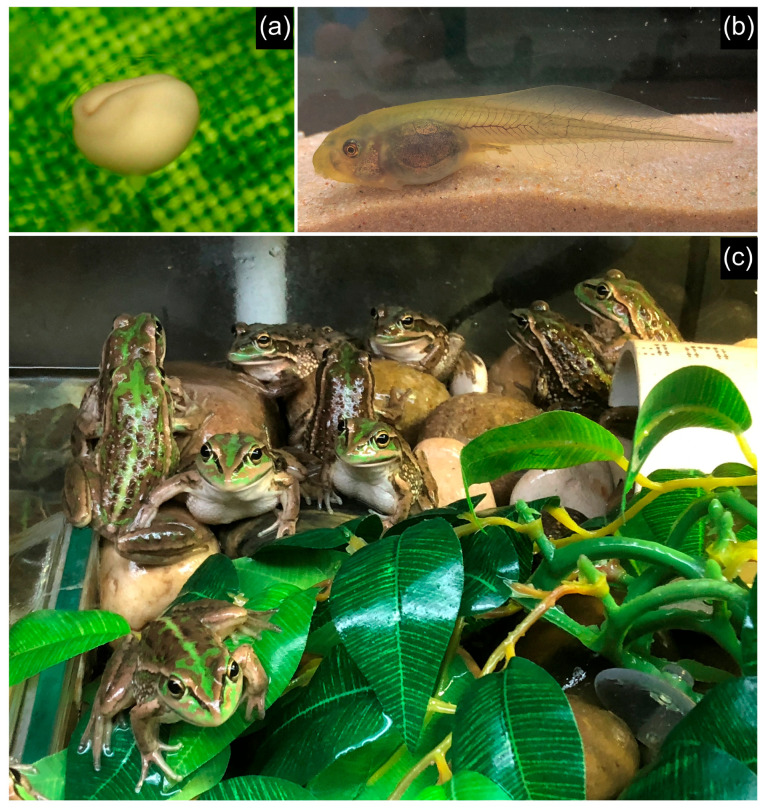
Study species, *R. castanea*: (**a**) an unpigmented egg, (**b**) a developed tadpole, and (**c**) recently metamorphosed frogs basking in an indoor enclosure.

In late 2024, breeding was attempted in the indoor conservation facility with first-generation zoo-bred frogs, consisting of pairs or trios, in nine standard enclosures. Breeding was very successful, with six clutches produced between 1 and 15 November 2024 (Table 2). Four of the clutches were produced from male and female *R. castanea*. These clutches numbered from 963 to 1168 unpigmented eggs (Figure 3a), with one clutch being infertile. The remaining two clutches were produced from pairings where both frogs were sibling backcrosses from the 2017 *R. castanea* and *R. r. raniformis* reproduction event. Although primarily unpigmented, the developing embryos developed a light grey colouration on day two.

Eggs typically took 3–4 days to hatch at a water temperature between 22 and 24 °C. For eggs laid in the large outdoor enclosure between 2017 and 2019, the fertility rate was estimated to be greater than ninety percent. For those produced in the 2024 breeding events, from the smaller subsets of eggs retained, the fertility rate was estimated to be approximately fifty percent. Tadpoles were maintained in the standard enclosures described previously, with mechanical and biological filtration, and water changes of approximately ten percent per day undertaken via an automated water change to the sumps. Aeration was provided to the sumps to minimise agitation of the water surrounding the tadpoles. Tadpoles were maintained on a diet of frozen endive and zucchini provided *ad libitum* and the daily addition of fish flakes, consisting of Sera Flora and Sera Sans flakes. Larval survival rates were relatively high, ranging from 81 to 92% for the tanks recorded, which is similar to those reported for *R. aurea* [18] and considerably higher than those reported for *R. moorei* [19].

Time to metamorphosis was largely determined by water temperature during larval rearing. For the 2017 clutches, at a temperature of 20–22 °C, metamorphosis first occurred from 74 days, with an average larval duration of 126 days and a small subset of tadpoles overwintering and metamorphosing in spring (Table 3). In 2024, at an increased water temperature of 22–24 °C, metamorphosis began much earlier, at 44 days post-hatching with the mean larval duration occurring at 74 days. The total tadpole length just prior to metamorphosis was much smaller for the 2017 clutches ($\bar{x}$ ± SEM = 65.10 ± 1.10 mm) compared to the 2024 clutches ($\bar{x}$ ± SEM = 81.9 ± 1.02 mm) (Figure 3b) (Table 3), which may have been due to the lower water temperature or greater larval rearing density. Both tadpole body length and total tadpole length were statistically different between the 2017 and 2024 tadpoles (*t*-tests: body length = *t*-ratio −0.246, df 48.238, *p*-value 0.807; total body length = *t*-ratio 0.56, df 38.998, *p*-value 0.579 *) (Table 3). However, the average length and mass of juveniles at the time of metamorphosis was almost identical in both years and not statistically different (*t*-tests: SVL length = *t*-ratio 6.915, df 46.813, *p*-value < 0.001*; body mass = *t*-ratio 11.301, df 46.901, *p*-value < 0.001*) (Table 3).

Juvenile frogs were fed daily on a diet of small *A. domestica*, dusted with the Repashy Calcium Plus calcium and multivitamin supplement. Juvenile frogs exhibited a very low mortality rate of less than 2% over the first three months. The male frogs matured and developed nuptial pads in the first season post-metamorphosis at approximately 8 months of age. From the offspring withheld in the insurance colony (i.e., not released into the wild), an even sex ratio was observed, with 11 males and 14 females from the pure *R. castanea* crosses, and 6 males and 5 females from the clutch produced from the *R. r. raniformis* female and *R. castanea* male hybrid mating.

### 3.2. Mortality

Unexpected mortality occurred in the early stages of the program, with six of the founder frogs dying prior to four years of age. Although a necropsy could not be performed on one due to autolysis, the remaining five frogs died from a combination of an emerging pathogen and renal disease. These five individuals, and one other that died just prior to five years of age, were infected with the myxozoan parasite *Cystodiscus axonis* [20]. The parasite caused extensive haemorrhagic lesions to the brain, spinal cord, and root nerve of the infected specimens. The individuals were infected with *C. axonis* upon collection from the wild, with two dying at under one year of age after being held in strict quarantine conditions. This was further confirmed when sampling from the wild collection site revealed significant levels of infection with *C. axonis* in eight out of ten Stony Creek Frogs (*Rhyaconastes wilcoxi*) and one out of five Eastern Banjo Frogs (*Limnodynastes dumerilii*) [21]. Both species were common at the site due to their lesser susceptibility to chytrid fungus. It is unclear if or how renal failure and subsequent edema is linked to *C. axonis*; however, these symptoms only presented in individuals infected with this parasite.

In May 2022, a mortality event impacted a small number of first-generation zoo-bred *R. castanea* when a chytrid fungus infection entered and spread within the large outdoor enclosure. On 29 May 2022, a dead frog was sighted in the open whilst the frogs were overwintering. An intensive search of the enclosure found three more recently deceased frogs and located a Striped Marsh Frog (*Limnodynastes peronii*), which subsequently tested positive for chytrid fungus. After being built six years earlier, slight ground movement had caused small gaps to form between the fencing panels and the frame, which resulted in the frog entering. The remaining four *R. castanea* were located and immediately treated for chytrid fungus via a shallow immersion in 0.01% suspension of itraconozole for 5 min daily for 6 days. Swabs later indicated that three of the four frogs tested positive for chytrid fungus infection at the time that treatment commenced. Two of the four frogs exhibited clinical symptoms of the disease and died whilst treatment was being administered, whilst the remaining two frogs survived and tested negative for chytrid fungus infection following treatment. After being deemed clear of chytrid infection, these frogs were returned to the indoor conservation facility.

## 4. Discussion

This paper reports on the establishment of the conservation breeding program and first successful breeding of *R. castanea* at Taronga Zoo, documenting clutch size and offspring measurement data. The breeding of this species was initially achieved in 2017, with a total of eleven clutches being produced to date. The calling period from late October to late December at the zoo is closely aligned with the core calling period observed in the rediscovered wild population of late November to late December [14]. The timing of reproduction, larvae size, and larvae behaviour were similar to closely related species within the Bell Frog species group, genus *Ranoidea* [22].

The average clutch size for those produced by first-generation frogs indoors was 1069 eggs, whilst the estimated average for the two clutches produced by founders in the outdoor enclosure was 1650 eggs. The clutches produced in the outdoor enclosure had a fertility and hatching rate of greater than ninety percent, whilst the fertility and hatching rate was substantially lower in a subset from the indoor facility, at approximately fifty percent. Overall, clutch sizes reported herein are lower than the previously documented data of two wild clutches for this species, containing 1885 and 3893 eggs, respectively [12]. The average clutch size is also lower than the average previously documented for *R. raniformis* of 3400 eggs [15]. The causes of the lower clutch size and lower fertility rate of zoo-bred individuals remain unclear, though this may be due to a number of factors including inbreeding depression, female size/age, female nutrition, or the ex situ environment. In contrast, data on the closely related *R. aurea* has found that the average clutch sizes of wild and zoo-bred individuals were quite similar, with 3773 and 3885 eggs reported for each, respectively [16,23].

A unique reproductive feature recorded in our study species is that every clutch observed has consisted of unpigmented eggs. The ova of the most closely related species, *R. raniformis*, typically have pigmented eggs with a black animal pole and white vegetal pole [22,24]. The hatching larvae of *R. raniformis* are also dark in colouration. In all clutches documented in this paper, both the eggs and hatching larvae were unpigmented. Larvae typically remained unpigmented for 3–4 days post-hatching before developing a very light grey colouration which progressed to brown colouration throughout development. It is not clear why there is a lack of pigmentation on the eggs of this species, as the breeding habits are very similar to both *R. aurea* and *R. raniformis*, including eggs being laid in submerged, gelatinous clumps in areas with exposure to sunlight. Amphibian eggs laid in this matter typically have melanin pigmentation on the dorsal hemisphere of the embryo to provide protection against exposure to damage by ultraviolet light [25]. Those species with unpigmented eggs typically deposit clutches in sheltered/concealed locations, such as subterranean nests, under rocks, or skin pouches [26].

Our findings indicate that rearing temperature influences the time to metamorphosis, with tadpoles reared at 22–24 degrees Celsius metamorphosing, on average, 52 days faster than tadpoles reared at 20–22 degrees. Tadpole length also differed, with tadpoles reared at the cooler temperature significantly smaller in size. Notably, tadpoles were only from a small number of clutches, and the clutches reared at the two different temperatures were genetically distinct (different parents). As such, we cannot eliminate the possibility that genetic factors contributed to the observed differences in time to metamorphosis or tadpole size, and further research investigating the effect of rearing temperature will need to be conducted to confirm the proximate mechanism for the result observed. Interestingly, our results showed that despite significant differences in the size of tadpoles, the length and mass of juvenile frogs at the time of metamorphosis was statistically similar and individual variation in size very low. These results are consistent with those observed in the Baw Baw frog, *Philoria frosti*, where tadpoles must reach a body-size threshold in order to trigger metamorphic onset [27]. Research in the Baw Baw frog has shown that rearing temperature and food availability influences time to metamorphosis, but that there was no effect on body mass or length [27]. Our observations similarly suggest that *R. castanea* tadpoles must reach a body-size threshold prior to metamorphosis and that, despite the large variation in tadpole size, individuals metamorphose at a similar size.

Although breeding was successfully attained and has now been repeated on multiple occasions, it is notable that this took a considerable amount of time. The first male frogs reached maturity during the 2011/12 season, with females reaching maturity in 2012/13. From this time, there were five seasons without reproductive success before spawning was achieved. During this period, breeding was attempted in various indoor and outdoor facilities whilst utilising a variety of techniques, including advertisement call playback and rain-simulating spray systems. The staff maintaining this species also had extensive experience breeding the closely related *R. aurea* in enclosures almost identical to those used for this species [16]. Despite employing the same techniques as for *R. aurea*, and observing amplexus on multiple occasions, attempts to attain oviposition from *R. castanea* in such indoor enclosures utilising the founder frogs were not successful. This demonstrates that it should not be automatically assumed that breeding will initially be successful, even if extensive experience has already been attained with an analogue species [28]. Thus, for declining species, efforts should always be made to establish conservation breeding protocols prior to the species requiring urgent intervention.

Successful reproduction was achieved after transferring the frogs to the large outdoor enclosure. Housing the frogs outdoors had many benefits, including the use of a large pond with a gradient of depths, live vegetation, natural climatic conditions, and plentiful opportunities to bask. One of these factors, or a combination of them, may have resulted in the successful breeding. The technique of large outdoor enclosures has also been effective for breeding in other conservation programs, such as the dusky gopher frog (*Lithobates sevosus*) [29]. Establishing smaller mesocosms in an outdoor environment after prior indoor attempts had failed has also been successful for the alpine tree frog (*Rawlinsonia verreauxii alpina*), further demonstrating the benefits of natural environmental triggers [30]. However, housing in outdoor enclosures also presents substantial risks, especially for Australian frogs, where the amphibian chytrid fungus remains a predominant driver of biodiversity loss. The transmissibility and lethality of chytrid fungus was demonstrated when a single wild infected frog entered the outdoor enclosure, resulting in the death of six of the eight frogs. Fortunately, a risk-averse strategy had been employed, with most of the population still being housed in an indoor, biosecure facility. Thus, only a smaller number of frogs were impacted by this mortality event.

Reproductive success for first-generation frogs was substantially improved compared to the founder individuals. In late 2024, six of the nine standard enclosures within the indoor conservation facility that had been set up for breeding, utilising six- or seven-year-old zoo-bred frogs, produced clutches of eggs. This rate of reproduction was similar to that experienced previously by zoo-bred *R. aurea* [16]. The enclosure conditions, environmental parameters, and husbandry were very similar to those utilised during early attempts to breed in the initial years of the program. It is possible that the zoo-bred individuals were more adapted to the ex situ environment and consequently had an increased readiness to breed in captive conditions. This explanation, however, seems unlikely, given that the founders, which exhibited poor breeding success, were collected at an early life stage (tadpoles) and reared under captive conditions until sexual maturity. An alternative explanation is that initial breeding success was compromised in our founder animals due to their infection with a myxozoan parasite, *Cystodiscus axonis*, confirmed in six of the founder animals. It has previously been demonstrated that Myxosporean parasites negatively impact reproduction in fish and amphibian species [31]. Thus, it is highly possible that reproduction in our founders was compromised due to parasitic infection obtained in the wild prior to collection. Fortunately, this parasite has not been transferred to the first-generation offspring of this species, with the ex situ environment likely missing an unknown intermediate host stage in the life cycle of the parasite [32].

This conservation breeding program also highlights the importance of disease surveillance of wild individuals at the time of collection to establish a baseline knowledge of in situ diseases and pathogens. In conservation breeding programs, disease screening prior to translocations is important to ensure that diseases or pathogens are not accidentally introduced to an already threatened wild population. However, background knowledge of the wild population is first required to know what pathogens may already be present in the wild and potentially present in the initial collected founders. In this program, six frogs died at under five years of age due to a myxosporean parasite. Fortunately, through the disease screening of common species sourced from the wild site at the time of collection, it was established that this pathogen was prevalent at the founder collection site. Thus, it could be determined that this pathogen likely entered the quarantine population with the founder frogs and should not prevent translocations from being undertaken. Furthermore, it has since been determined that the pathogen was not transmitted between individuals within the ex situ environment, likely due to the lack of an intermediate host. The importance of baseline disease screening of wild populations has previously been highlighted in other amphibian translocation programs [33], including for the endangered Booroolong frog (*Rhyaconastes booroolongensis*), where a myxosporean parasite was also found in the brains of a number of individuals of the species at the time of collection [34].

Also noteworthy was the successful hybrid mating between *R. castanea* and *R. r. raniformis*, and for the first-generation pairings between these offspring to produce fertile young. This supports the close genetic relationship identified between these taxa, where the taxonomic status requires further clarification [13]. Such reproduction between closely related pelodrayadid taxa, producing fertile hybrids and back-crossed offspring, has recently been documented for *R. booroolongensis* with the Stony Creek Frog (*R. wilcoxii*) [35]. In our study species, although hybrid reproduction occurred between the taxa on at least one occasion, there was also evidence for mate selection, avoiding hybridisation in the large outdoor enclosure in favour of pure matings within *R. castanea*. From the three fertile clutches laid throughout 2017 and 2018, genetic testing showed that the female laying the eggs was of the *R. castanea* taxon on two occasions, and of the *R. r. raniformis* taxon on one occasion. Thus, fifty percent of breeding opportunities with female *R. castanea* resulted in breeding success, whilst only six percent of opportunities were successful with *R. r. raniformis*. This result was not unexpected, as interspecies hybridisation has been previously documented in closely related frog species, but with hybridisation observed at much lower rates than intraspecies matings [36]. The successful hybrid mating between *R. castanea* and *R. r. raniformis* potentially has important implications for the long-term genetic management of *R. castanea*. As only a small number of founding animals were able to be collected from the wild population, the risk of the further erosion of genetic diversity in the insurance colony over time is a concern. To avoid inbreeding depression, the introgression of genetic diversity from hybrid matings between *R. castanea* and *R. r. raniformis* may be considered as part of the long-term genetic management of the species.

The successful breeding detailed in this paper is significant as the success of the conservation breeding program will largely determine the future of this species. With the exception of individuals reintroduced into the wild from the conservation breeding program, this species has not been detected in the wild since 2014, despite extensive searches. Thus, its future persistence relies on the production of zoo-bred offspring and translocation efforts. To further enhance breeding efforts and create a genetic safeguard for the species, a sperm cryopreservation protocol has been established, with sperm biobanked from all founders and a proportion of the F1 individuals. Further to this, an assisted fertilisation program is being initiated to generate offspring from genetically under-represented individuals. The biobanked sperm will also be used to extend the reproductive lifespan of male individuals through the introgression of expired genotypes, which will be critical for the long-term maintenance of genetic diversity. The rediscovered population was wiped out by stochastic climatic events and disease, and to-date, experimental translocations of 2162 juvenile frogs to three sites have been hampered by climatic conditions, including flood events and severe drought. Future efforts will focus on achieving reliable breeding protocols and developing a successful translocation program by identifying suitable release sites and producing reliable output of offspring for reintroduction.

## 5. Conclusions

Our study provides an overview of the conservation breeding program for the critically endangered *R. castanea*, detailing the breeding challenges and successes during the initial establishment of an insurance colony for this species. Despite only having breeding success in large outdoor enclosures with the initial founder frogs, we found that the first-generation zoo-bred frogs reproduced more readily in indoor enclosures. We were able to establish a number of life history parameters for the species, such as the key breeding period from October to December, and data on clutch size, larval duration, and tadpole and metamorph sizes. The successful breeding events, spanning four breeding seasons, have resulted in the release of 2162 juvenile frogs into the wild.

Importantly, the present study highlights a few key learnings. One of these is that there should be urgency in establishing programs for species with critically low numbers. Fortunately, this was implemented with this species prior to its disappearance from the wild. Another is that despite having great success breeding an analogue species, it cannot be automatically assumed that this will translate to a closely related species. In this instance, it took a number of years, and the trialing of several different techniques, to successfully breed *R. castanea*. Finally, we underscore the importance of undertaking disease screening in wild populations to determine a baseline of parasites and pathogens present in the wild. We anticipate that the insights gained from the present study will assist in highlighting important considerations for the establishment of conservation breeding programs for threatened species globally.

## Figures and Tables

**Table 1:** Summary of *R. castanea* reproductive attempts between 2011/12 and 2016/17.

Year	Calling Recorded	No. of Frogs (Male/Female)	Amplexus Observed	Facility Utilised	Additional Breeding Tools Utilised
2011/12	21 October–12 November 2011	6/6	Three pairs between 31 October–12 November 2011.	Standard indoor enclosure in initial quarantine	Call playback from late October to late December
2012/13	15 November–31 December 2012	3/3	Four occasions between 16 November 2012 and 1 January 2013.	Standard indoor enclosure in conservation facility	8 November 2012–31 January 2013—Call playback of male advertisement call played at various times throughout night. 6 December 2012–31 January 2013—Irrigation spray system utilised intermittently, especially during natural rain events.
	None	2/2	No	Large indoor enclosure in conservation facility.	
2013/14	28 November–1 December 2013	3/3	One pair on 28 November 2013 only.	Large indoor enclosure in conservation facility.	4 November–20 December 2013—Call playback
	None	2/2	No	Suspended outdoor enclosure	21 December 2013–25 January 2014—Call playback
2014/15	15 November–25 December 2014	5/4	One pair observed on 24 November 2014 and 1 January 2015	Suspended outdoor enclosure	
2015/16	No calling recorded	5/2	No	Suspended outdoor enclosure	
2016/17	27 October–10 November 2016	5/2	No	Large outdoor enclosure	

**Table 2:** Clutches of *R. castanea* produced at Taronga Zoo between 2017 and 2024 in the various enclosure types.

Year	Facility Utilized	No. of Frogs (M/F)	Generation	Clutch	Clutch Date	Clutch Size
2017/18	Large outdoor enclosure	5/2 (+0.8 *R. r. raniformis*)	Founders	1	12 November 2017	1500 ± 100
				2	12 November 2017	1800 ± 100
2018/19	Large outdoor enclosure	4/2 (+0.8 *R. r. raniformis*)	Founders	3	13 November 2018	unknown
2019/20	Large outdoor enclosure	4/2 (+0.7 *R. r. raniformis*)	Founders	4	24 October 2019	unknown
				5	1 November 2019	unknown
2024/25	Standard indoor enclosure	9/6 (*R. castanea*)	F1	6	1 November 2024	1032
				7	3 November 2024	1168
				8	3 November 2024	963
				9	16 November 2024	1112
	Standard indoor enclosure	6/4 (*R. castanea*/*R. r. raniformis* cross)	F1	10	1 November 2024	1698
				11	15 November 2024	unknown

**Table 3:** Offspring development; the average and range of the larval period (time from hatching to metamorphosis), larval size, and metamorph size for a subset of the offspring produced in 2017 and 2024.

Year	Water Temp (°C)	Larval Period (Days)	Larval Body Length (mm)	Larval Total Length (mm)	Metamorph SVL Length (mm)	Metamorph Mass (g)
2017	20–22	126 (74–316)	27.56 ± 0.46 * (22.9–31.7)	65.10 ± 1.10 * (56.2–72.3)	27.14 ± 0.39 (21.6–31.4)	1.87 ± 0.09 (0.8–3.1)
2024	22–24	74 (44–118)	32.14 ± 0.48 * (27.5–36.7)	81.93 ± 1.02 * (72.4–92.6)	27.03 ± 0.28 (25.0–28.5)	1.94 ± 0.09 (1.4–2.4)

Data presented are for 25 tadpoles and 44 metamorphs across 2 clutches in 2017, and 24 tadpoles and 12 metamorphs across 2 clutches in 2024. Data shown are mean ± SEM (range; min–max). * denotes statistical significance between years (*t*-tests, *p* < 0.05; JMP Pro 19.0 statistical software).

## Data Availability

The breeding data presented in this study are available on request from the corresponding author. The data are not publicly available, in accordance with Taronga Conservation Society Australia’s policies on data and sample sharing (Opportunistic Sample Request Policy). Additional information on the microsatellite loci, primer sequences, and amplification conditions used by the Australian Museum to determine maternity are available on request to the corresponding author.

## Abbreviations

The following abbreviations are used in this manuscript:

EPBC	Environmental Protection and Biodiversity Conservation
IUCN	International Union for the Conservation of Nature
NSW	New South Wales
UVB	Ultraviolet B
PVC	Polyvinyl Chloride
